# A Novel Derivative of Curcumol, HCL-23, Inhibits the Malignant Phenotype of Triple-Negative Breast Cancer and Induces Apoptosis and HO-1-Dependent Ferroptosis

**DOI:** 10.3390/molecules28083389

**Published:** 2023-04-12

**Authors:** Peng Zhao, Hui Song, Futian Gao, Liang Chen, Jianfei Qiu, Jun Jin, Chaolan Pan, Yunyan Tang, Meijun Chen, Yang Pan, Yanmei Li, Liejun Huang, Jue Yang, Xiaojiang Hao

**Affiliations:** 1 State Key Laboratory of Functions and Applications of Medicinal Plants & Key Laboratory of Endemic and Ethnic Diseases & Ministry of Education & Key Laboratory of Medical Molecular Biology of Guizhou Province, Guizhou Medical University, Guiyang 550025, China; 2 The Key Laboratory of Chemistry for Natural Products of Guizhou Province and Chinese Academic of Sciences, Guiyang 550014, China; 3 School of Pharmaceutical Sciences, Guizhou Medical University, Guiyang 550025, China; 4 School of Pharmaceutical Sciences, Guizhou University, Guiyang 550025, China; 5 Key Laboratory of Modern Pathogen Biology and Characteristics, School of Basic Medicine, Guizhou Medical University, Guiyang 550025, China; 6 Research Unit of Chemical Biology of Natural Anti-Virus Products, Chinese Academy of Medical Sciences, Beijing 100730, China

**Keywords:** triple-negative breast cancer, curcumol derivative, metastasis, apoptosis, ferroptosis

## Abstract

Triple-negative breast cancer (TNBC) is the most aggressive molecular subtype of breast cancer. Curcumol, as a natural small molecule compound, has potential anti-breast cancer activity. In this study, we chemically synthesized a derivative of curcumol, named HCL-23, by structural modification and explored its effect on and underlying mechanism regarding TNBC progression. MTT and colony formation assays demonstrated that HCL-23 significantly inhibited TNBC cells proliferation. HCL-23 induced G2/M phase cell cycle arrest and repressed the capability of migration, invasion, and adhesion in MDA-MB-231 cells. RNA-seq results identified 990 differentially expressed genes including 366 upregulated and 624 downregulated genes. Gene Ontology (GO), Kyoto Encyclopedia of Genes and Genomes (KEGG), and Gene Set Enrichment Analysis (GSEA) revealed that these differentially expressed genes were obviously enriched in adhesion, cell migration, apoptosis, and ferroptosis. Furthermore, HCL-23 induced apoptosis via the loss of mitochondrial membrane potential and the activation of the caspase family in TNBC cells. In addition, HCL-23 was verified to trigger ferroptosis through increasing cellular reactive oxygen species (ROS), labile iron pool (LIP), and lipid peroxidation levels. Mechanistically, HCL-23 markedly upregulated the expression of heme oxygenase 1 (HO-1), and the knockdown of HO-1 could attenuate ferroptosis induced by HCL-23. In animal experiments, we found that HCL-23 inhibited tumor growth and weight. Consistently, the upregulation of Cleaved Caspase-3, Cleaved PARP, and HO-1 expression was also observed in tumor tissues treated with HCL-23. In summary, the above results suggest that HCL-23 can promote cell death through activating caspases-mediated apoptosis and HO-1-dependent ferroptosis in TNBC. Therefore, our findings provide a new potential agent against TNBC.

## 1. Introduction

Cancer remains a leading cause of death worldwide. In females, breast cancer has become the most common cancer globally, with the highest incidence (24.5%) and mortality (15.5%) [1]. Breast cancer can be clinically divided into three main subtypes, including human epidermal growth factor receptor 2 (HER2)-positive, hormone receptors (HR)-positive, and triple-negative breast cancer (TNBC) [2]. Due to tumor heterogeneity and lacking known therapeutic targets, patients with TNBC showed the most unfavorable outcomes and high rates of recurrence [3]. Although adjuvant chemotherapy has significantly improved the survival rate, chemotherapeutic drugs are still clinically associated with low selectivity, and patients who received long-term chemotherapy will eventually develop to drug resistance [4]. Therefore, the development of novel TNBC chemotherapy drugs has become an urgent clinical need.

Most anti-cancer drug therapy prevents tumor growth by inducing apoptosis, which is characterized by an endoplasmic reticulum and/or mitochondrial expansion [5]. However, chemoresistant cancer cells usually gain the ability to escape apoptosis. In order to solve this problem, researchers began to focus on other kinds of programmed cell death (PCD), such as autophagy, pyroptosis, and ferroptosis. Ferroptosis is a new kind of PCD that is triggered by increased iron and lipid peroxidation [6]. In recent studies, ferroptosis has been broadly implicated in various cancers, including liver, colon, and stomach cancer [7,8,9]. For instance, tamoxifen-resistant breast cancer cells are more vulnerable to erastin-induced ferroptosis [10]. Metformin can induce ferroptosis by inhibiting SLC7A11 UFMylation in breast cancer [11]. This suggests that the activation of ferroptosis could serve as a potential treatment strategy for breast cancer.

Curcumol, a major sesquiterpene hemiacetal containing a ring system derived from *Curumae Rhizoma* [12], shows a wide range of pharmacological activities, such as anti-inflammatory [13], anti-viral [14], and anti-tumor activites. In breast cancer, curcumol inhibits the metastasis of MDA-MB-231 and 4T1 cells through JNK1/2- and AKT-dependent NF-κB signaling pathways and increases the sensitivity of TNBC to doxorubicin by regulating the miR-181b-2-3p-ABCC3 axis [15,16]. In addition, curcumol can induce the apoptosis of MDA-MB-231 cells by activating p-73 and PUMA [17]. Taken together, these findings suggest that curcumol has potential anti-breast cancer activity. Nevertheless, its poor water solubility and low bioavailability seriously hinder the clinical application prospect of curcumol.

**Figure 1:**
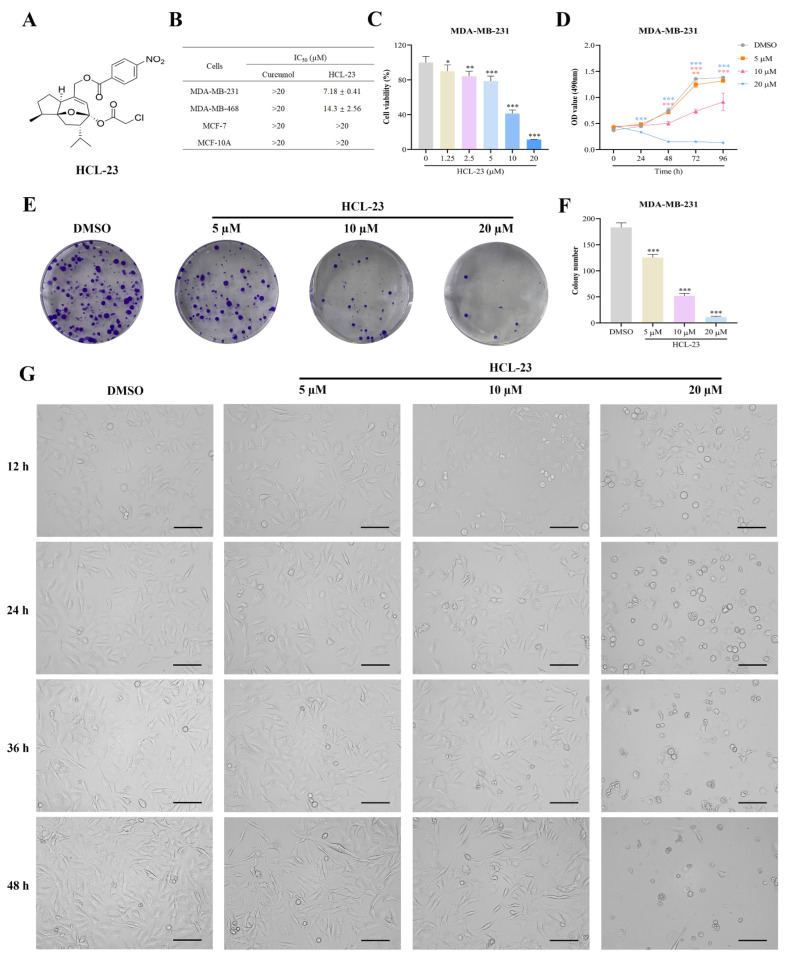
HCL-23 inhibits cell proliferation in MDA-MB-231 cells (**A**) Chemical structure of HCL-23. (**B**) The IC_50_ values of HCL-23 in the human breast cancer cell lines MDA-MB-231, MDA-MB-468 and MCF-7 and the human normal mammary epithelial cell line MCF-10A at 72 h. (**C**) MDA-MB-231 cells were treated with different concentrations of HCL-23 (1.25, 2.5, 5, 10, and 20 µM) for 72 h. The cell viability was measured using an MTT assay. (**D**) The effect of HCL-23 on MDA-MB-231 cells proliferation was determined using an MTT assay. (**E**) MDA-MB-231 cell colonies were visualized after treatment with HCL-23 (5, 10, and 20 µM) for 14 days. (**F**) Bar graph was used to quantify the number of visible colonies. (**G**) Representative images of MDA-MB-231 cells morphology after treatment with different concentrations of HCL-23 (5, 10, and 20 µM) at 12, 24, 36, and 48 h, (magnification ×200, scale bar: 100 μm). Data represent the mean ± SD of three independent experiments. * *p* < 0.05, ** *p* < 0.01, *** *p* < 0.001 vs. the DMSO group.

Recently, chemical structure modification has become an effective strategy for improving its druggability. In this study, we chemically synthesized a derivative of curcumol, 3-O-(chloroacetyl)-9-O-(p-nitrobenzoyl)-curcumol (HCL-23, Figure 1A), by structural modification and assessed its anti-breast cancer activity in vitro in different breast cancer cell lines. In addition, the effects of HCL-23 on the biological function were measured by colony formation, wound healing, transwell, cell cycle, and apoptosis assays. RNA-seq was used to analyze the potential mechanism. Finally, 4T1 tumor-bearing mice were applied to evaluate the effect of HCL-23 on tumor growth in vivo.

## 2. Results

### 2.1. HCL-23 Inhibits Cell Proliferation and Induces G2/M Phase Cycle Arrest in MDA-MB-231 Cells

**Scheme 1:**
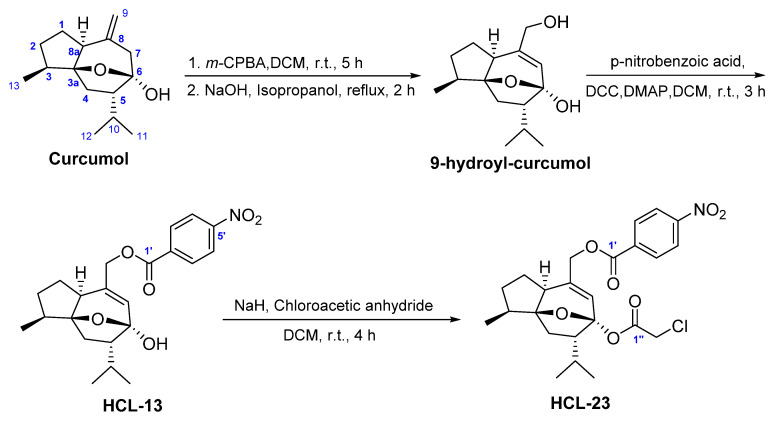
The synthetic route of compounds HCL-13 and HCL-23.

**Figure 2:**
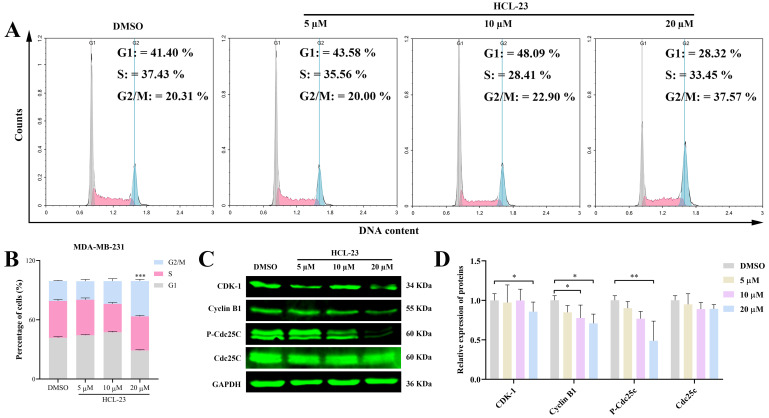
HCL-23 induces G2/M phase cell cycle arrest in MDA-MB-231 cells. (**A**) Cell cycle phase distribution of MDA-MB-231 cells treated with various concentrations of HCL-23 (5, 10, and 20 µM) for 12 h. (**B**) Quantification of cell cycle distribution at G1, S, and G2 phases. (**C**) MDA-MB-231 cells were treated with various concentrations of HCL-23 (5, 10, and 20 µM) for 12 h. Cell cycle-related proteins were analyzed by Western blot. (**D**) Quantification of cell cycle-related proteins. Data represent the mean ± SD of three independent experiments. * *p* < 0.05, ** *p* < 0.01, *** *p* < 0.001 vs. the DMSO group.

A series of curcumol derivatives, including seven derivatives we have previously reported [18] and, additionally, two newly synthesized derivatives (HCL-13 and HCL-23) (Scheme 1), have been screened for the inhibition of breast cancer cell lines using an MTT assay. Only one compound, HCL-23 (Figure 1A), showed an inhibitory effect on cell viability (Supplementary Table S1). Then, we further evaluated the anti-breast cancer activity of HCL-23 and curcumol in several human breast cancer cell lines by an MTT assay. The results showed that MDA-MB-231 cells were more sensitive to HCL-23, with an IC_50_ value of 7.18 ± 0.41 µM (Figure 1B). We also observed a better anti-tumor effect of HCL-23 compared to that of curcumol. We selected MDA-MB-231 cells for further study. In addition, HCL-23 decreased MDA-MB-231 cell viability in a dose-dependent manner (Figure 1C). Through a cell growth curve and a colony formation assay, we observed an inhibitory effect of HCL-23 on MDA-MB-231 cell proliferation in a concentration- and time-dependent manner (Figure 1D–F). At 12, 24, 36, and 48 h, microscopic observation showed that HCL-23 decreased the cell number and exhibited shattered and irregular morphology (Figure 1G). We also tested the influence of HCL-23 on the cell cycle. As shown in Figure 2A,B, HCL-23 treatment at 20 μM caused G2/M phase cell cycle arrest in MDA-MB-231 cells. Western blot results confirmed that HCL-23 downregulated the levels of cell cycle-related proteins, such as CDK-1, Cyclin B1, and P-Cdc25C (Figure 2C,D).

### 2.2. HCL-23 Suppresses the Migration, Invasion, and Adhesion of MDA-MB-231 Cells

**Figure 3:**
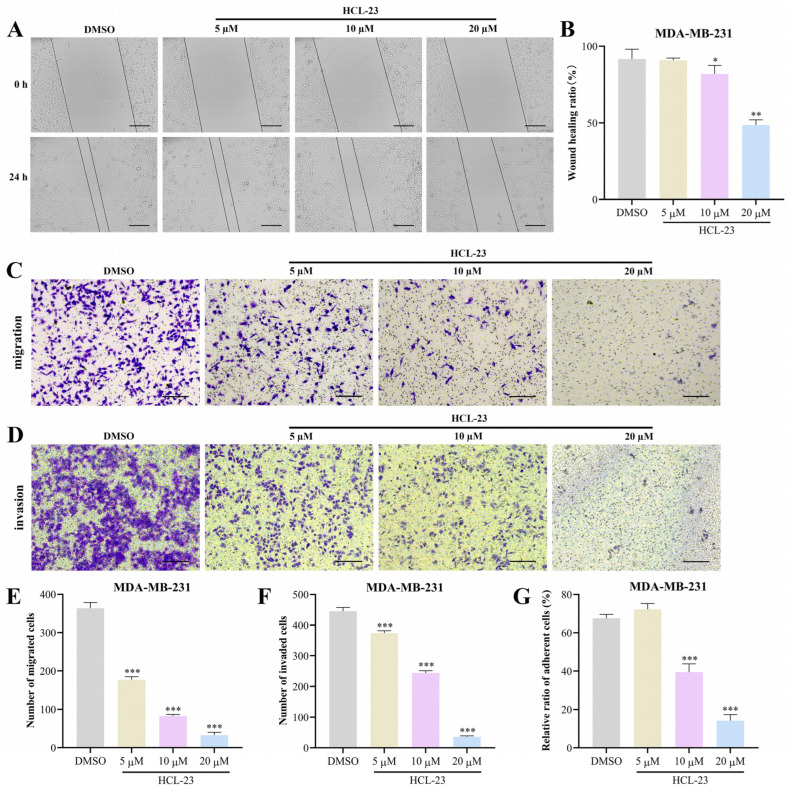
HCL-23 suppresses the migration, invasion, and adhesion of MDA-MB-231 cells. (**A**) Representative images of wound healing assays of MDA-MB-231 cells treated with various concentrations of HCL-23 (5, 10, 20 µM) for 0 h and 24 h (magnification ×100, scale bar: 200 μm). (**B**) Quantification of wound healing ratio (%). (**C**) Transwell migration assays of MDA-MB-231 cells treated with various concentrations of HCL-23 (5, 10, and 20 µM) for 24 h (magnification ×100, scale bar: 200 μm). (**D**) Transwell invasion assays of MDA-MB-231 cells treated with various concentrations of HCL-23 (5, 10, and 20 µM) for 24 h (magnification ×100, scale bar: 200 μm) (**E**) Quantification of the number of migrated cells. (**F**) Quantification of the number of invaded cells. (**G**) Effect of various concentrations of HCL-23 (5, 10, and 20 µM) on the cell adhesion of MDA-MB-231 cells for 24 h. Data represent the mean ± SD of three independent experiments. * *p* < 0.05, ** *p* < 0.01, *** *p* < 0.001 vs. the DMSO group.

To investigate the effect of HCL-23 on the metastasis of MDA-MB-231 cells, we performed wound healing and transwell assays. As shown in Figure 3A,B, HCL-23 significantly inhibited the ability of TNBC cells to close a gap in a wound healing assay. The transwell assay showed that the number of migrated (Figure 3C,E) and invaded (Figure 3D,F) cells in the HCL-23 treatment group was dramatically decreased compared with that of the DMSO group. Furthermore, the inhibitory tendency was enhanced with the increasing concentration of HCL-23. In the meantime, the cell adhesion assay revealed that HCL-23 can markedly reduce the capability of cell adhesion (Figure 3G). The above results demonstrated that HCL-23 inhibited TNBC cell metastasis in a concentration-dependent manner.

### 2.3. RNA-Seq Analysis of HCL-23-Related Biological Processes and Pathways

**Figure 4:**
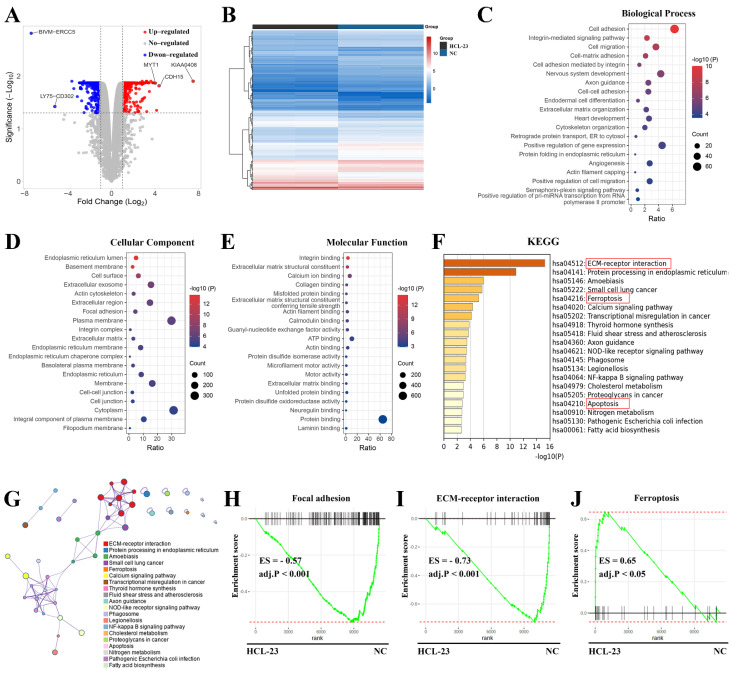
RNA-seq analysis of HCL-23-related biological processes and pathways. (**A**) Volcano plot shows the differentially expressed genes between MDA-MB-231 cells treated with and without HCL-23. Red dots represent upregulated genes, blue dots represent downregulated genes, and grey dots represent unchanged genes. Differentially expressed genes were identified according to the criteria: |log2FC| > 1 and adj *p* < 0.05. (**B**) Heat map of differentially expressed genes in cells treated with or without HCL-23. (**C**–**E**) Bubble plot shows the top 20 enriched terms of differentially expressed genes for the biological process (**C**), cellular component (**D**), and molecular function (**E**), which were analyzed by DAVID. (**F**) The top 20 enriched KEGG pathways of differentially expressed genes were analyzed by Metascape. (**G**) Interaction network of the top 20 enriched KEGG pathways. (**H**–**J**) GSEA was conducted to evaluate the influence of HCL-23 on KEGG pathways by Networkanalyst 3.0. Focal adhesion (**H**). ECM–receptor interaction (**I**). Ferroptosis (**J**).

To explore the anti-proliferative mechanism of HCL-23 on the MDA-MB-231 cells, RNA-seq was applied to evaluate the influence of HCL-23 on gene transcription. As shown in Figure 4A,B, 990 differentially expressed genes were identified, including 366 upregulated and 624 downregulated genes. As expected, biological process analysis showed that 990 differentially expressed genes were strongly related to cell adhesion, cell migration, and cell–matrix adhesion (Figure 4C). Cellular component analysis revealed that these differentially expressed genes were enriched in the endoplasmic reticulum lumen, focal adhesion, and extracellular matrix (Figure 4D). Molecular function analysis indicated that HCL-23 was significantly associated with the integrin binding, extracellular matrix structural constituent, and collagen binding function (Figure 4E). KEGG enrichment analysis showed that HCL-23 might exert its anti-breast cancer effect by regulating several pathways, such as ECM–receptor interaction, ferroptosis, and apoptosis (Figure 4F,G). Compared to the DMSO group, the GSEA results indicated that the enrichment scores of focal adhesion and ECM–receptor interaction were strikingly downregulated, while the enrichment score of ferroptosis was significantly upregulated with HCL-23 treatment (Figure 4H–J). These findings suggested that HCL-23 might inhibit TNBC cell proliferation by inducing apoptosis and ferroptosis.

### 2.4. HCL-23 Induces Apoptosis in MDA-MB-231 Cells

**Figure 5:**
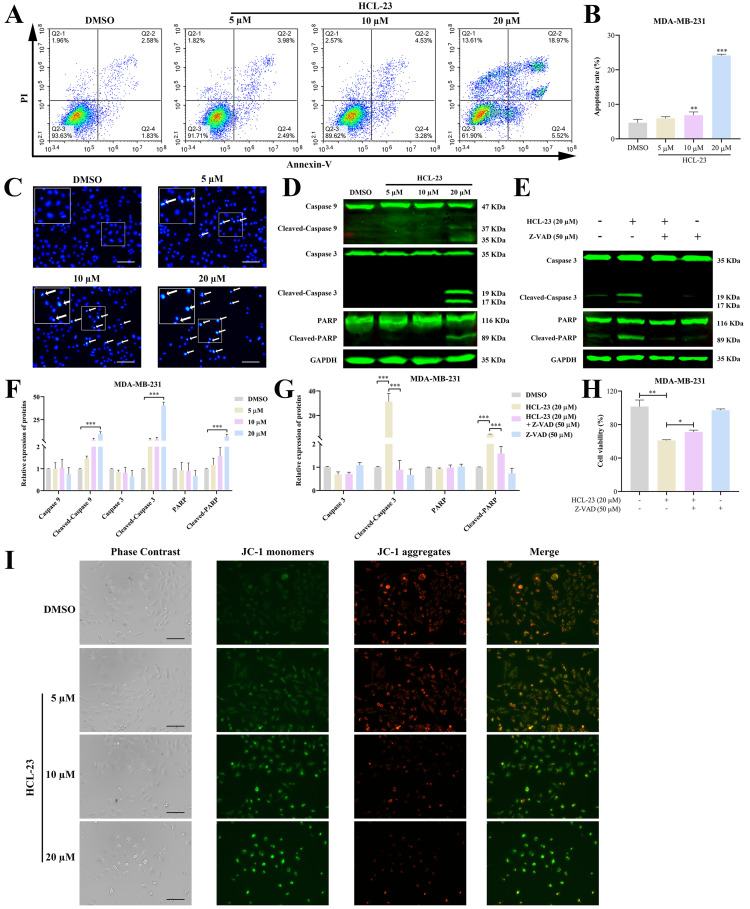
HCL-23 induces apoptosis in MDA-MB-231 cells. (**A**) Annexin V-FITC/PI double staining was used to assess the apoptosis of MDA-MB-231 cells induced by HCL-23 (5, 10, and 20 μM) for 24 h. (**B**) Quantification of the apoptosis percentage. (**C**) Representative fluorescence images of MDA-MB-231 cells stained with Hoechst 33258 after treatment with different concentrations (5, 10, and 20 μM) of HCL-23 for 24 h (the white arrow indicates apoptotic cells). (**D**) MDA-MB-231 cells were treated with different concentrations (5, 10, and 20 μM) of HCL-23 for 24 h. The expression levels of Caspase 9, Cleaved-Caspase 9, Caspase 3, Cleaved-Caspase 3, PARP, and Cleaved-PARP were analyzed by Western blot. (**E**) HCL-23 (20 µM) upregulated the protein expression of Cleaved-Caspase 3 and Cleaved-PARP in MDA-MB-231 cells, which could be blocked by Z-VAD-FMK (50 µM). (**F**) Quantification of apoptosis-related proteins after treatment with different concentrations of HCL-23. (**G**) The quantification of apoptosis-related proteins after treatment with HCL-23 (20 µM) and/or Z-VAD (50 µM). (**H**) Z-VAD-FMK (50 µM) treatment partially reversed the inhibitory effect of HCL-23 (20 µM) on cell viability in MDA-MB-231 cells. (**I**) MDA-MB-231 cells were treated with HCL-23 for 24 h and then incubated with JC-1 staining buffer. Mitochondrial membrane potential was examined by fluorescence microscopy (magnification ×200; scale bar 100 μm). Data represent the mean ± SD of three independent experiments. * *p* < 0.05, ** *p* < 0.01, *** *p* < 0.001.

As the RNA-seq result indicated a potential involvement of HCL-23 in apoptosis and ferroptosis, we first explored how HCL-23 influenced apoptosis in TNBC cells. Flow cytometry analysis showed that HCL-23 can markedly induce MDA-MB-231 cells apoptosis in a concentration-dependent manner (Figure 5A,B). In the meantime, as shown in Figure 5C, HCL-23 treated MDA-MB-231 cells displayed apoptotic morphological characteristics, including nuclear shrinkage and chromatin condensation via Hoechst 33258 staining. Western blot results confirmed that different concentrations of HCL-23 treatment significantly upregulated the expression of the apoptosis-related proteins Cleaved-Caspase 9, Cleaved-Caspase 3, and Cleaved-PARP (Figure 5D,F). Pretreatment with pan-Caspase inhibitor Z-VAD-FMK (Z-VAD) attenuated HCL-23-mediated cell death as well as the levels of Cleaved-Caspase 3 and Cleaved-PARP in MDA-MB-231 cells (Figure 5E,G,H). JC-1 fluorescence analysis showed that green fluorescence intensity was obviously enhanced with the increasing concentration of HCL-23, suggesting that HCL-23 can decrease the mitochondrial membrane potential of MDA-MB-231 cells (Figure 5I). In summary, treatment with HCL-23 inhibited the growth of TNBC cells via apoptosis, which might be correlated with the loss of mitochondrial membrane potential and the activation of the caspase family.

### 2.5. HCL-23 Triggers Ferroptosis in MDA-MB-231 Cells

**Figure 6:**
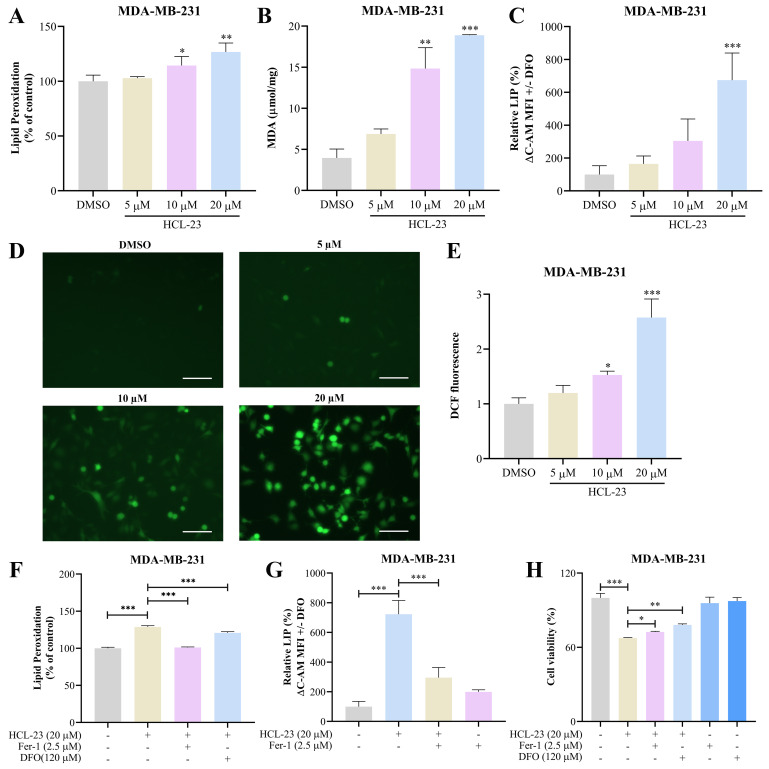
HCL-23 triggers ferroptosis in MDA-MB-231 cells. (**A**) Treatment with HCL-23 for 12 h increased the level of lipid peroxidation in MDA-MB-231 cells in a concentration-dependent manner. (**B**) Treatment with HCL-23 for 12 h increased the level of MDA. (**C**) Treatment with HCL-23 for 12 h increased the level of LIP. (**D**) MDA-MB-231 cells were treated with HCL-23 for 12 h and then incubated with DCFH-DA probe. ROS level was examined by fluorescence microscopy (magnification ×200; scale bar 100 μm). (**E**) DCF fluorescence intensity was quantified by flow cytometry. (**F**) HCL-23 (20 µM) increased the level of lipid peroxidation in MDA-MB-231 cells, which could be blocked by Fer-1 (2.5 µM) and DFO (120 µM). (**G**) Fer-1 (2.5 µM) treatment eliminated the increased level of LIP induced by HCL-23 (20 µM) in MDA-MB-231 cells. (**H**) HCL-23 (20 µM) decreased the cell viability in MDA-MB-231 cells, but Fer-1 (2.5 µM) and DFO (120 µM) counteracted HCL-23-induced inhibition of cell viability. Data represent the mean ± SD of three independent experiments. * *p* < 0.05, ** *p* < 0.01, *** *p* < 0.001.

Subsequently, we investigated whether HCL-23 could induce ferroptosis in MDA-MB-231 cells, as inferred above by KEGG analysis. Lipid peroxidation levels in MDA-MB-231 cells were increased by HCL-23 at 10 μM and 20 μM (Figure 6A). As shown in Figure 6B, HCL-23 increased Malondialdehyde (MDA) levels in a concentration-dependent manner. Compared with the DMSO group, the levels of LIP and ROS were higher with HCL-23 treatment, especially in the high-dose group (Figure 6C–E). The enhancement of lipid peroxidation and LIP induced by HCL-23 was partially alleviated with the treatment of ferroptosis inhibitor ferrostatin-1 (Fer-1) [19] and deferoxamine (DFO) [20] (Figure 6F,G). More importantly, we further confirmed whether the inhibitory effect of HCL-23 on cell viability was mediated by ferroptosis. Our data illustrated that Fer-1 and DFO could abate the decrease in MDA-MB-231 cell viability due to HCL-23 treatment (Figure 6H).

### 2.6. HCL-23 Induces Ferroptosis by Up-Regulating HO-1 Expression

**Figure 7:**
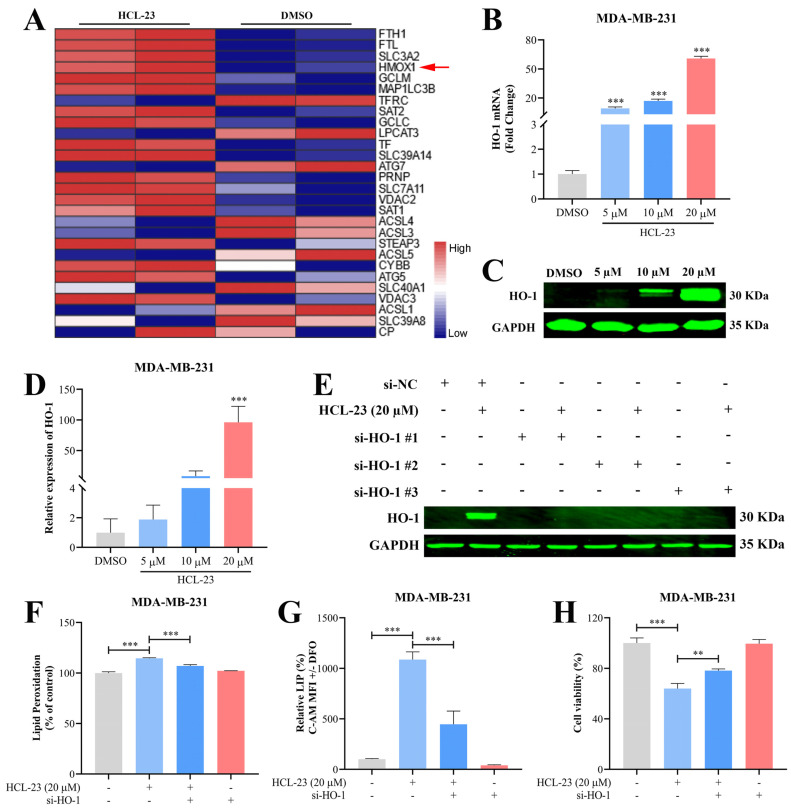
HCL-23 induces ferroptosis by up-regulating HO-1 expression. (**A**) Heat map of ferroptosis-related genes in response to HCL-23 treatment using RNA-seq analysis. Low expression is depicted in blue, and high expression is depicted in red. (**B**) The mRNA level of HO-1 was measured by qRT-PCR after treatment with different concentrations of HCL-23 for 24 h. (**C**) The protein level of HO-1 was analyzed by Western blot in MDA-MB-231 cells treated with HCL-23 for 24 h. (**D**) Quantification of HO-1 protein expression. (**E**) Western blot was used to detect the protein level of HO-1 in MDA-MB-231 cells treated with HCL-23 and transfected with specific siRNA for HO-1. (**F**) HCL-23 (20 µM) increased the level of lipid peroxidation in MDA-MB-231 cells, which could be partly neutralized by si-HO-1 #1 transfection. (**G**) HO-1 knockdown reversed the increased level of LIP induced by HCL-23 (20 µM) in MDA-MB-231 cells. (**H**) si-HO-1 counteracted HCL-23-induced inhibition of cell viability. Data represent the mean ± SD of three independent experiments. ** *p* < 0.01, *** *p* < 0.001 vs. the DMSO group.

In order to explore the possible mechanism of HCL-23-induced ferroptosis, we further analyzed the transcriptional alteration of ferroptosis-related genes. RNA-seq results showed that HO-1 expression was significantly upregulated in MDA-MB-231 cells after HCL-23 treatment (Figure 7A). A growing number of studies have demonstrated that high HO-1 expression is a trigger of iron overload-induced ferroptosis [21,22]. Consistent with the RNA-seq results, the qPCR results showed that the mRNA level of HO-1 was upregulated 10-fold with 5 μM, 15-fold with 10 μM, and 60-fold with 20 μM after HCL-23 treatment for 24 h (Figure 7B). Western blot validated that HCL-23 remarkably increased the protein level of HO-1 (Figure 7C,D). We preformed specific siRNA for HO-1 to investigate whether HO-1 participated in HCL-23-induced ferroptosis in MDA-MB-231 cells. si-HO-1 transfection reversed the increase in HO-1 induced by HCL-23 (Figure 7E). In addition, the upregulated level of lipid peroxidation and LIP caused by HCL-23 was reduced after HO-1 knockdown (Figure 7F,G). Then, we further confirmed whether the inhibitory effect of HCL-23 on cell viability was related to HO-1. Our data showed that HO-1 knockdown could attenuate the decrease in MDA-MB-231 cell viability caused by HCL-23 treatment (Figure 7H). These results revealed that HCL-23 inducing ferroptosis was mediated by upregulating HO-1 expression in MDA-MB-231 cells.

### 2.7. HCL-23 Inhibits Breast Tumor Growth In Vivo

**Figure 8:**
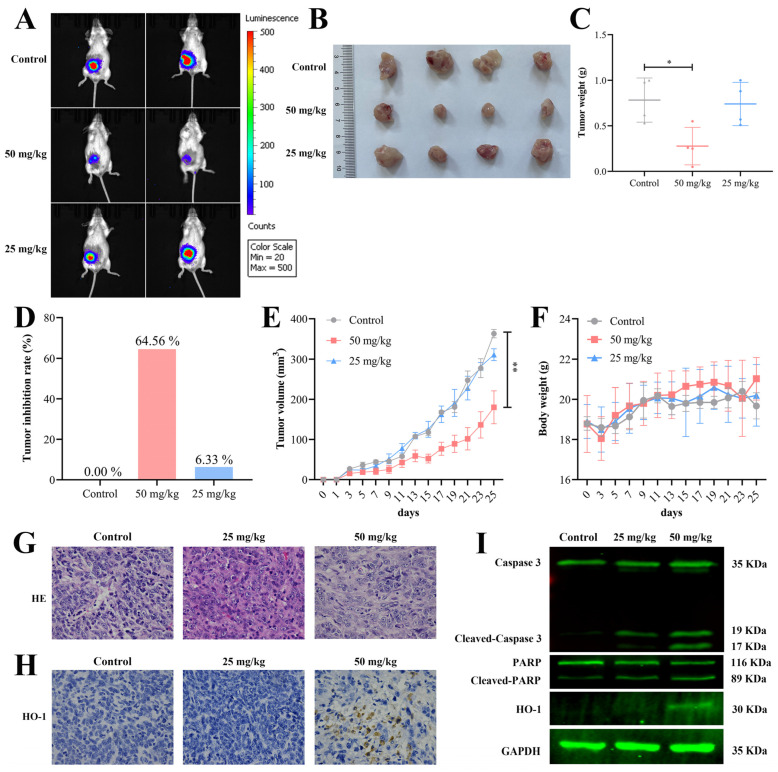
HCL-23 inhibits breast tumor growth in vivo. Mouse breast cancer 4T1-Luc cells were orthotopically injected into female BALB/c mice and treated with HCL-23 (50 mg/kg and 25 mg/kg) (n = 4/group). (**A**) Representative images of tumors formed in mice were visualized by a live imaging system. (**B**) The images of tumors at the endpoint of the experiment. (**C**) Tumor weight from different groups. (**D**) Tumor inhibition rate (%). (**E**) Tumor growth curve. (**F**) Mice body weight curve. (**G**) Representative images of HE staining (magnification ×400). (**H**) Representative images of IHC staining for HO-1 (magnification ×400). (**I**) The expression levels of Caspase 3, Cleaved-Caspase 3, PARP, Cleaved-PARP, and HO-1 in tumor tissues were measured by Western blot. Data represent the mean ± SD. * *p* < 0.05, ** *p* < 0.01 vs. the control group.

To further validate the anti-breast tumor effect of HCL-23 in vivo, 4T1-Luc cells were subcutaneously injected into the breast fat pad of female BALB/c mice to establish a TNBC animal model. The results showed that 50 mg/kg of HCL-23 could significantly attenuate the bioluminescent signal of tumor sites (Figure 8A). Compared to those in the control group, the administration of 50 mg/kg of HCL-23 markedly reduced the tumor weight and volume (Figure 8B–E). However, there was no obvious impact on the body weight of HCL-23 (Figure 8F). HE staining showed that nuclear division and the ratio of the nucleus to the cytoplasm were decreased after HCL-23 treatment (Figure 8G). The IHC results verified that 50 mg/kg of HCL-23 treatment markedly upregulated the expression of HO-1 (Figure 8H). Consistently, the Western blot results showed that the expression levels of Cleaved-Caspase 3, Cleaved-PARP, and HO-1 were significantly increased in 50 mg/kg of the HCL-23 group (Figure 8I).

## 3. Discussion

**Figure 9:**
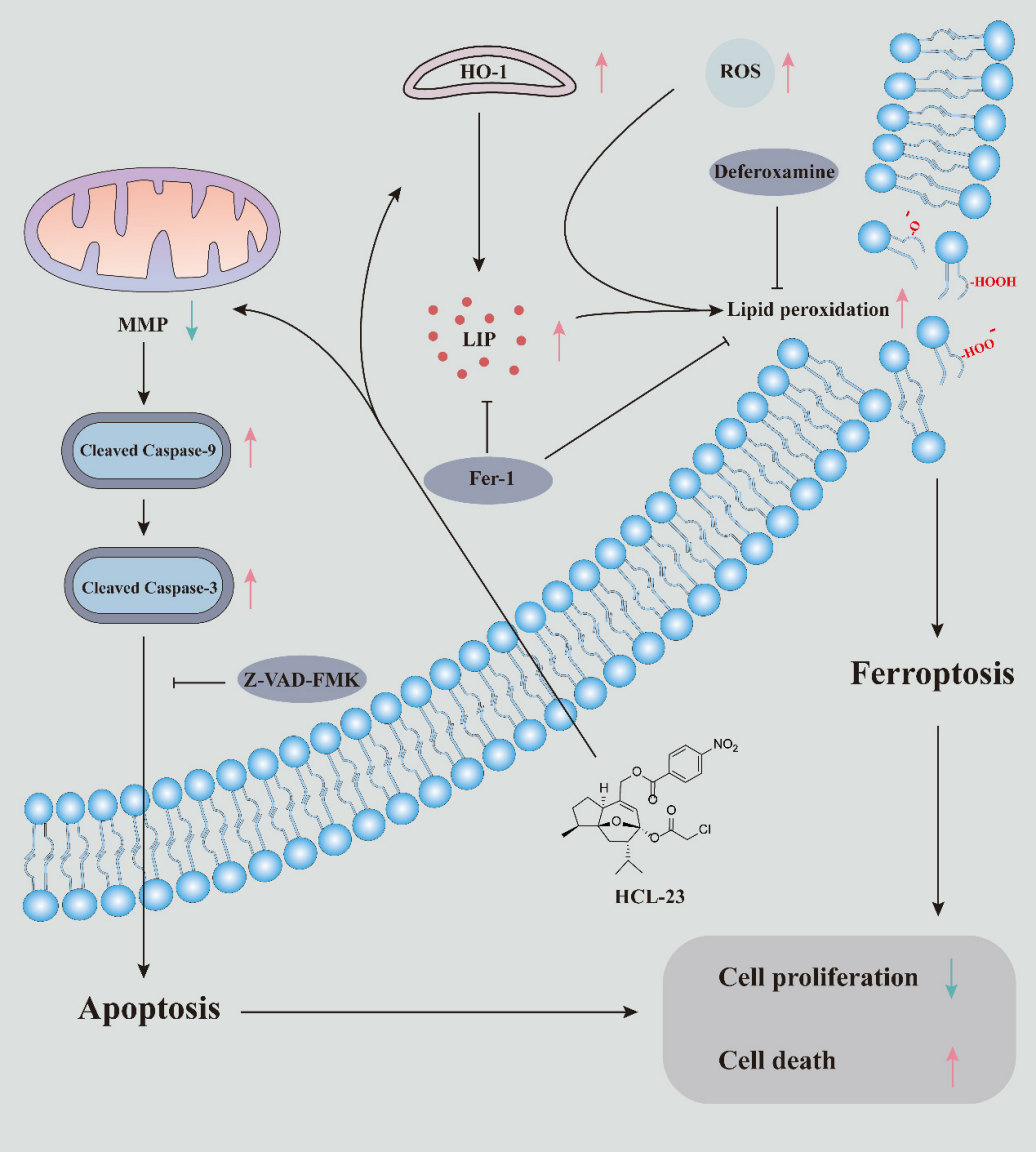
HCL-23 induces caspases-mediated apoptosis and HO-1-dependent ferroptosis in MDA-MB-231 cells.

At present, natural small molecule compounds are still important sources for the discovery of anti-tumor lead compounds and drug development [23]. As an effective new drug development strategy, the structural modification of natural small molecule compounds with anti-tumor potential can obtain more druggable compounds. In this study, we synthesized a derivative of curcumol, HCL-23, which showed a better anti-TNBC effect than curcumol. Notably, we found that HCL-23 not only inhibited cells metastasis and caused G2/M phase cell cycle arrest, but it also induced two main types of PCD: apoptosis and ferroptosis. These effects were closely associated with the loss of mitochondrial membrane potential, the activation of the caspase family, labile iron overload, the generation of ROS, and the upregulation of HO-1 (Figure 9).

It is well known that TNBC is the most metastatic and aggressive molecular subtype of breast cancer. The median overall survival of patients with metastatic TNBC is approximately 18 months due to the lack of an effective therapeutic target [24]. Metastasis is a complex, multi-step biological process including the loss of intercellular contacts, the invasion of the extracellular matrix, spreading, and detachment events. These processes are involved with multiple signal pathways [25]. Our results showed that HCL-23 can significantly inhibit TNBC cells metastasis. Interestingly, RNA-seq analysis of HCL-23-treated cells revealed that 990 differentially expressed genes were enriched in several metastasis-related biological processes and signal pathways, such as cell migration, cell–matrix adhesion, focal adhesion, and ECM–receptor interaction. It is reported that focal adhesion is involved in various cellular processes, such as cell adhesion, migration, and proliferation [26]. Emerging studies have indicated that focal adhesion kinase (FAK), a key regulator of focal adhesion dynamics, can regulate breast cancer cell adhesion and metastatic dissemination through signal transduction by integrins and other cell surface receptors [27,28].

In this study, KEGG pathway analysis revealed that the inhibitory effect of HCL-23 on the cell viability of breast cancer may be mediated by two types of PCD: apoptosis and ferroptosis. Previous studies have revealed that the activation of the caspase cascade plays a critical role in apoptotic mechanisms [29,30]. The intrinsic or mitochondrial pathway can activate caspases, which is associated with a loss of mitochondrial membrane potential [31,32,33,34]. Consistently, our results demonstrated that HCL-23 can induce apoptosis through activating caspase 9/caspase 3 and decreasing the mitochondrial membrane potential while arresting the cell cycle at the G2/M phase by reducing the protein levels of CDK-1, Cyclin B1, and P-Cdc25C. Moreover, the cytotoxic effect of HCL-23 on TNBC cells was partially reduced by treatment with a pan-caspase inhibitor (Z-VAD-FMK). It can be inferred that apoptosis is one of the pathways of death in our study. It is usually considered that apoptosis is an important mechanism of many anti-tumor drugs. However, an increasing number of studies have shown that long-term drug use will cause cancer cells to escape apoptosis, resulting in chemotherapy resistance [35,36]. Therefore, targeting other kinds of PCD has also become a new strategy for anti-tumor drug development.

As another important kind of PCD, growing evidence has shown that ferroptosis can be an alternative for killing apoptosis-resistant cancer cells [37,38]. Several FDA-approved drugs, such as sorafenib, sulfasalazine, and artemisinin, can improve the curative effect of chemotherapy drugs through inducing tumor cell ferroptosis [39]. Ferroptosis depends on LIP, lipid peroxidation, and ROS [40]. MDA is one of the products of lipid peroxidation, which is an indicator of ferroptosis [41]. Interestingly, we found that HCL-23 could increase the levels of ROS, LIP, MDA, and lipid peroxidation in MDA-MB-231 cells in a concentration-dependent manner. Specifically, the increased levels of lipid peroxidation and LIP induced by HCL-23 were partially reversed by the ferroptosis inhibitor Fer-1 and the iron chelator DFO. In addition, Fer-1 and DFO also alleviated the inhibitory effect of HCL-23 on cell viability. These findings suggested that HCL-23 was a potential inducer of ferroptosis in TNBC cells.

HO-1, a key enzyme for heme degradation, plays an important role in cardiovascular disease, glucose metabolism, and tumor development [42,43]. It is reported that HO-1 is increased in a variety of malignant tumors, including renal cancer, hepatocellular carcinoma, and pancreatic cancer [44]. Upregulated HO-1 acted as an oncogene to promote cancer cell growth, metastasis, and resistance to anticancer therapy [45,46]. However, several studies elucidated a controversial role of HO-1 in tumor development. For instance, Shuganning injection could inhibit TNBC cell proliferation by inducing HO-1-dependent ferroptosis [47]. In breast cancer, Li et al. found that the inhibition of HO-1 reduced curcumin-induced ferroptosis [48]. These above-mentioned findings highlighted a complex function of HO-1 in breast cancer, and understanding its detailed role might help to shed light on the relationship between oxidation and tumorigenesis. Utilizing RNA-seq, we identified HO-1 as a candidate downstream target of HCL-23. Strikingly, qPCR, Western blot, and IHC staining validated that HCL-23 treatment dramatically upregulated the expression of HO-1. Furthermore, HO-1 knockdown weakened the increasing levels of lipid peroxidation, LIP, and inhibition on cell viability induced by HCL-23 in MDA-MB-231 cells, indicating that HCL-23 inducing ferroptosis is in an HO-1-dependent manner. This is consistent with Du and Li’s reports that upregulating HO-1 is an important mechanism of LIP accumulation and ferroptosis [47,48].

In summary, we found that a novel curcumol derivative, HCL-23, exerted excellent anti-TNBC activity in inhibiting cell proliferation and metastasis, inducing cell cycle arrest, apoptosis, and ferroptosis both in vitro and in vivo. Furthermore, HCL-23 promoted cell death through activating caspases-mediated apoptosis and HO-1-dependent ferroptosis. Overall, this study highlighted HCL-23 as a promising potential agent against TNBC. Although the anti-breast cancer activity of HCL-23 is stronger than that of curcumol, further investigation is needed to evaluate its solubility, bioavailability, and stability via in vitro and in vivo experiments and further improve its druggability by structural modifications.

## 4. Materials and Methods

### 4.1. Chemistry

Curcumol was isolated from the oil of *Oleum curcumae* (purchased from local commercial sources) [18]. Reagents and solvents were purchased from Adamas, JK chemical, and local commercial sources. The reagents and the solvents were purified according to the guidelines in Purification of Laboratory Chemicals. ^1^ H and ^13^ C NMR spectra were recorded on a Bruker Avance NEO 600 spectrometer with TMS as the internal standard. ESI-MS data were carried out on an Agilent 1100 instrument and Thermo ultimate 3000/Q EXACTIVE FOCUS mass spectrometers. Column chromatography was obtained on silica gel (40–80, 200–300, 300–400, mesh; Qingdao Marine Chemical Co. Ltd., Qingdao, China). Fractions were monitored by TLC (GF_254_, Qingdao Marine Chemical Co., Ltd., Qingdao, China), and spots were detected with a UV254 lamp via heating SiO_2_ plates sprayed with 7% H_2_SO_4_ in EtOH.

### 4.2. Chemical Structure Information of HCL-13 and HCL-23

#### 4.2.1. HCL-13

White powder; Yield 67% ^1^H NMR (600 MHz, Acetone-*d*_6_) δ 8.38–8.40 (2H, dt, *J* = 8.8, 1.9 Hz, H-4′, 6′), 8.29–8.31 (2H, dt, *J* = 8.8, 1.9 Hz, H-3′, H-7′), 6.00 (1H, s, H-7), 5.35 (1H, s, OH), 4.83–4.91 (2H, m, H-9), 1.00–1.01(3H, d, *J* = 6.6 Hz, H-11), 0.97–1.01(3H, d, *J* = 6.6 Hz, H-12), 0.86–0.88 (3H, d, *J* = 6.6 Hz, H-13); ^13^C NMR (150 MHz, Acetone-*d*_6_) *δ*: 164.1 (C-1′), 150.8 (C-5′), 137.7 (C-8), 135.6 (C-2′), 130.7 (C-3′, 7′), 128.9 (C-7), 123.7 (C-4′, 6′), 103.0 (C-6), 85.9 (C-3α), 66.8 (C-9), 59.1 (C-5), 49.6 (C-8α), 40.1 (C-3), 36.2 (C-4), 31.3 (C-2), 30.8 (C-10), 27.5 (C-1), 22.3 (C-11), 21.0 (C-12), 11.2 (C-13); ESI-MS *m*/*z*: 424.1 [M+Na]^+^. (calcd for C_22_H_27_NO_6_, 401.1.) (Supplementary Figures S1–S3).

#### 4.2.2. HCL-23

White powder; Yield 49% ^1^H NMR (600 MHz, Acetone-*d*_6_) *δ* 8.38–8.40 (2H, d, *J* = 8.9 Hz, H-4′, 6′), 8.30–8.32 (2H, d, *J* = 8.9 Hz, H-3′, H-7′), 6.17 (1H, s, H-7), 4.88–4.94 (m, 2H, H-9), 4.26–4.32 (2H, m, H-2′), 1.00–1.01 (3H, d, *J* = 6.6 Hz, H-11), 0.97–0.98 (3H, d, *J* = 6.6 Hz, H-12), 0.91–0.92 (3H, d, *J* = 6.6 Hz, H-13); ^13^C NMR (150 MHz, Acetone-*d*_6_) *δ* 164.1 (C-1′, 1′′), 150.8 (C-5′), 136.7 (C-8), 135.6 (C-2′), 130.7 (C-3′, 7′), 126.2 (C-7), 123.7 (C-4′, C-6′), 106.2 (C-6), 89.1 (C-3a), 66.7 (C-9), 57.8 (C-5), 49.4 (C-8a), 41.3 (C-2′′), 39.8 (C-3), 34.5 (C-4), 31.0 (C-2), 30.2 (C-10), 27.4 (C-1), 21.9 (C-11), 20.8 (C-12), 11.1 (C-13); ESI-MS *m*/*z*: 500.0 [M+Na]^+^. (calcd for C_24_H_28_O_7_NCl, 477.0.) Purity of HCL-23 ≥ 95%. (Supplementary Figures S4–S7).

### 4.3. Cell Culture

Mouse breast cancer 4T1-Luc cells labeled with luciferase were purchased from Mingjing Biology (Shanghai, China). The human breast cancer cell lines MDA-MB-231, MDA-MB-468, and MCF-7 and the human normal mammary epithelial cells MCF-10A were purchased from the American Type Culture Collection (ATCC, Manassas, VA, USA). All cells were cultured in DMEM (Hyclone) containing 10% fetal bovine serum (FBS, GIBCO) and 1% Penicillin-Streptomycin Solution (Hyclone, Logan, UT, USA) under a 5% CO_2_ and humidified atmosphere at 37 °C.

### 4.4. Cell Viability Assay

The cell viability was assessed by an MTT assay according to a previous description [49]. Briefly, 6 × 10^3^ cells per well were seeded into 96-well plates. After culturing overnight, the cells were treated with different concentrations of HCL-23 for the indicated time. Then, 10 µL 3-(4,5-dimethylthiazol-2-yl)-2,5-diphenyl-2H-tetrazolium bromide (MTT, 5 mg/mL) was added to each well and incubated for 4 h at 37 °C. The supernatant was moved, and the precipitate was dissolved in dimethyl sulfoxide (DMSO). The absorbance was subsequently measured at 490 nm using a spectrophotometer (BioTek, Winooski, VT, USA). The IC_50_ was calculated using the relative survival curve.

### 4.5. Colony Formation Assay

MDA-MB-231 cells were seeded into a six-well plate at a density of 500 cells per well. Then, cells were treated with different concentrations of HCL-23. After being cultured for 14 days, the cells were fixed with methanol for 30 min and stained with crystal violet solution (Solarbio, Beijing, China) for 15 min. The cells were then washed three times with PBS, and the number of clones was counted.

### 4.6. Cell Cycle and Apoptosis Assay

MDA-MB-231 cells (2 × 10^5^) were seeded into a six-well plate and treated with different concentrations of HCL-23 (5, 10, and 20 μM). For cell cycle analysis, the cells were fixed in 70% ethanol overnight at −20 °C, stained with propidium iodide (PI, BD Biosciences, Franklin lakes, NJ, USA) in the dark for 30 min at 37 °C, and then analyzed by a flow cytometer (BD Biosciences, Franklin lakes, NJ, USA). For cell apoptosis analysis, the cells were stained with FITC-Annexin V (BD Biosciences, Franklin Lakes, NJ, USA) and PI in the dark for 15 min at room temperature and then immediately analyzed by flow cytometry.

### 4.7. Wound-Healing Assay

MDA-MB-231 cells (8.0 × 10^5^) were seeded in a six-well plate and cultured overnight. A wound on each well was scratched in a straight line using a 10 µL pipette tip and then washed with PBS three times to remove non-adherent cells. Next, the serum-free medium containing different concentrations of HCL-23 was added and cultured for 24 h. Images were captured by microscopy at 0 h and 24 h, and the scratch area was quantified by Image J (National Institutes of Health, Bethesda, MD, USA).

### 4.8. Transwell Invasion and Migration Assay

The transwell chamber was used to examine the effect of HCL-23 on cell migration and invasion. For the cell invasion assay, Matrigel (BD Biosciences, Franklin lakes, NJ, USA) was used to coat the upper chambers at 100 µL per well. The lower chamber was filled with 600 µL of DMEM containing 15% FBS. A total of 100 µL of serum-free DMEM with 5 × 10^4^ HCL-23-treated MDA-MB-231 cells was seeded in the upper wells. The capacity of migration was assessed following the same procedure as the invasion assay, except the upper chamber was pre-coated without Matrigel. After 24 h, invaded and migrated cells were fixed with methanol, stained with 0.1% crystal violet solution, and counted by microscopy.

### 4.9. Cell Adhesion Assay

The treated cells were seeded in 96-well plates coated with Matrigel at a density of 5.0 × 10^4^ cells and cultured at 37 °C for 1 h. Then, the culture medium of non-adherent cells was discarded. A total of 100 µL of serum-free medium was added to each well, and then 10 µL of MTT solution was added to each well at 37 °C for 4 h. The supernatant of each well was discarded, and the crystal was immediately dissolved in 160 µL of DMSO per well and then measured at 490 nm with a microplate reader.

### 4.10. RNA-Seq and Functional Enrichment Analysis

The treated cells were collected and the total RNA was extracted using the TRIzol reagent (Invitrogen, Carlsbad, CA, USA). Transcriptome sequencing was performed by BGI Tech (Shenzhen, China) using the DNBSEQ platform. The raw data were filtered with SOAPnuke, and then mapped to the GRCh38.p13 using HISAT and Bowtie2. Differentially expressed genes were analyzed by the Limma R package using NetworkAnalyst 3.0 [50]. The threshold of differentially expressed genes was set as |log2 fold change| > 1 and adj. *p* < 0.05. Gene Ontology (GO, including the biological process, cellular component, and molecular function) and Kyoto Encyclopedia of Genes and Genomes (KEGG) analysis of differentially expressed genes were carried out by DAVID and Metascape, respectively [51,52]. Gene set enrichment analysis (GSEA) was conducted with NetworkAnalyst 3.0. The genes were ranked by Moderated Welch’s t-test, and the analysis mode was chosen as multi-level, as recommended.

### 4.11. Hoechst 33258 Staining

MDA-MB-231 cells (2.0 × 10^5^) were seeded in six-well plates and treated with different concentrations of HCL-23 for 24 h. The cells were washed twice with PBS and then stained with 0.5 mL Hoechst 33258 (Beyotime, Jiangsu, China) for 5 min. Images were captured by a fluorescence microscope (Leica Microsystems, Wetzlar, Germany).

### 4.12. Mitochondrial Membrane Potential Assay

MDA-MB-231 cells (2.0 × 10^5^) were seeded in six-well plates and treated with different concentrations of HCL-23 for 24 h. Then, the cells were stained with 1 mL JC-1 staining solution (Beyotime, Jiangsu, China) at 37 °C. After 20 min, the cells were washed twice with 1 × JC-1 staining buffer and observed under a fluorescence microscope.

### 4.13. Measurement of Reactive Oxygen Species (ROS)

DCFH-DA (Beyotime, Jiangsu, China) was used to detect ROS levels according to the manufacturer’s procedures. Briefly, the 2.0 × 10^5^ cells were seeded in six-well plates. After treatment with different concentrations of HCL-23, the cells were labeled with serum-free medium containing DCFH-DA in the dark at 37 °C for 20 min and observed under a fluorescence microscope. The fluorescence intensity of DCF was measured by flow cytometry.

### 4.14. Measurement of Lipid Peroxidation

The total cellular lipid peroxidation was measured using a C11 BODIPY (581/591) probe (GLPBIO, Montclair, NJ, USA). The cells were treated as indicated and incubated for 30 min at 37 °C with C11 BODIPY (1 µM) in fresh medium. Then, the cells were harvested and immediately analyzed by flow cytometry.

### 4.15. Measurement of Malondialdehyde (MDA)

An MDA Assay Kit (Beyotime, Nantong, China) was used to measure the cell MDA content. Briefly, MDA-MB-231 cells were seeded in six-well plates and treated with different concentrations of HCL-23. Then, the cells were collected and lysed. After protein quantification, MDA working solution was added and heated at 100 °C for 15 min. Next, the supernatant was collected through centrifuging at 15,000 rpm for 10 min at 4 °C and then measured at 532 nm using a microplate reader.

### 4.16. Determination of Labile Iron Pool (LIP)

The total LIP was detected by the calcein-ethoxymethyl ester (C-AM) method. The HCL-23-treated MDA-MB-231 cells were seeded in six-well plates, followed by incubation with 1 μM calcein-acetoxymethyI ester (GLPBIO, Montclair, NJ, USA) at 37 °C for 30 min. Then, the cells were harvested and treated with or without deferoxamine (120 µM) for 1 h at 37 °C and then analyzed by flow cytometry. The level of LIP was calculated according to the average fluorescence difference of the cells with and without deferoxamine incubation.

### 4.17. Western Blot Analysis

MDA-MB-231 cells and homogenized tumor tissues were lysed on ice with lysate buffer for 1 h. Protein samples (50 µg) were separated by SDS-PAGE and transferred to PVDF membranes. The membranes were blocked with 3% bovine serum albumin at room temperature for 1 h and then incubated with primary antibodies against CDK-1, Cyclin B1, Cdc25C, P-Cdc25C, Caspase 9, Caspase 3, PARP, and HO-1, which were obtained from the Abcam (Cambridge, UK) and CST (Danvers, MA, USA) overnight at 4 °C. The membranes were washed and incubated with FITC-labeled secondary antibody at room temperature for 2 h. Finally, the membranes were scanned using the Odyssey Platform (LI-COR Biosciences, Lincoln, NE, USA).

### 4.18. Quantitative Real-Time PCR (qPCR)

The treated cells were harvested, and the total RNA was extracted with the TRIzol reagent. The total RNA was reverse-transcribed to cDNA using the PrimeScript RT reagent Kit with the gDNA Eraser (TaKaRa, Beijing, China). Determinations of mRNA levels were performed using specific primers (Sangon Biotech, Shanghai, China) and LightCycler Multiplex Masters (Roche, Tucson, AZ, USA) in a StepOne Plus thermal cycler (Applied Biosystems, Carlsbad, CA, USA). GAPDH was used as an endogenous DMSO. Sequences of qPCR primers are listed in Supplementary Table S2.

### 4.19. siRNA Transfection

Specific small interfering RNA (siRNA) for HO-1 (si-HO-1) and its negative control (si-NC) were synthesized by Genepharma (Shanghai, China). Transfection was performed using Lipofectamine 3000 (Invitrogen, Carlsbad, CA, USA), according to the manufacturer’s instructions. The sequences of si-HO-1 were as follows:

si-HO-1#1 sense: 5′-GUAGGGCUUUAUGCCAUGUTT-3′,

si-HO-1#1 antisense: 5′-ACAUGGCAUAAAGCCCUACTT-3′,

si-HO-1#2 sense: 5′-CCAGCAACAAAGUGCAAGATT-3′,

si-HO-1#2 antisense: 5′-UCUUGCACUUUGUUGCUGGTT-3′,

si-HO-1#3 sense: 5′-GCUGAGUUCAUGAGGAACUTT-3′,

si-HO-1#3 antisense: 5′-AGUUCCUCAUGAACUCAGCTT-3′.

### 4.20. In Vivo Experiment

4T1-Luc cells (1 × 10^6^) were orthotopically injected into the mammary fad of 6-week-old female BALB/c mice. Then, 25 mg/kg and 50 mg/kg HCL-23 were intraperitoneally injected (n = 4/group). The body weight and tumor size were measured every other day. The tumor volume was calculated using the formula volume (mm^3^) = 1/2 × a × b^2^, where a and b are the long and short dimensions of the tumor, respectively. After D-Luciferin sodium salt (Yeasen, Shanghai, China) was injected, the IVIS Spectrum Living Imaging system (PerkinElmer, Waltham, MA, USA) was used to detect tumor bioluminescence intensity in mice. The tumor weight was recorded when the mice were sacrificed. All procedures were approved by the Animal Care and Use Committee of Guizhou Medical University (2101008).

### 4.21. Histology and Immunohistochemistry

The tumors isolated from the mice were fixed with 4% paraformaldehyde, embedded in paraffin, and sectioned to 4 µm slides for hematoxylin and eosin (HE) staining and immunohistochemistry (IHC). For the IHC assay, the slides were dehydrated with xylene and hydrated with ethanol. After antigen retrieval, the slides were blocked with 1% bovine serum albumin, stained with the primary antibody against HO-1 at 4 °C overnight, and incubated with the secondary antibody for 1 h, followed by staining with DAB and hematoxylin. Finally, the slides were observed under a microscope.

### 4.22. Statistical Analysis

All data were presented as the mean ± standard deviation (SD) from at least three independent experiments. Statistical analysis was performed using Student’s *t*-test or one-way ANOVA in GraphPad Prism 8.0 (GraphPad Software, La Jolla, CA, USA) and SPSS 26.0 (IBM Corporation, Armonk, NY, USA). *p <* 0.05 was considered statistically significant.

## Data Availability

The data presented in this article are available.

## Supplementary Materials

The following supporting information can be downloaded at: https://www.mdpi.com/article/10.3390/molecules28083389/s1, Figure S1: 600 MHz 1H NMR Spectrum of HCL-13 in acetone-*d*_6_; Figure S2: 150 MHz 13C NMR Spectrum of HCL-13 in acetone-*d*_6_; Figure S3: ESI-MS of HCL-13; Figure S4: 600 MHz 1H NMR Spectrum of HCL-23 in acetone-*d*_6_; Figure S5: 150 MHz 13C NMR Spectrum of HCL-23 in acetone-*d*_6_; Figure S6: ESI-MS of HCL-23; Figure S7: HR-ESI-MS of HCL-23; Table S1: IC_50_ values of curcumol derivatives in breast cancer; Table S2: The sequences of primers used in this study.
