# Supplementary material for: A Novel Derivative of Curcumol, HCL-23, Inhibits the Malignant Phenotype of Triple-Negative Breast Cancer and Induces Apoptosis and HO-1-Dependent Ferroptosis
Source: Molecules. 2023 Apr 12;28(8):3389. doi: 10.3390/molecules28083389 (PMC10142363; doi:10.3390/molecules28083389)
Supplement: Supplementary file 1 [file molecules-28-03389-s001.zip › molecules-2277346-supplementary.pdf]

Supplementary Table S1. IC_{50} values of curcumol derivatives in breast cancer.

Compound	Chemical structure	IC_{50} (μM)	
		MCF-7	MDA-MB-231
HCL-13	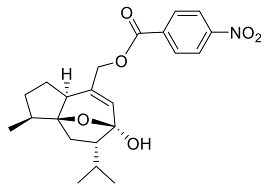	>20	>20
HCL-23	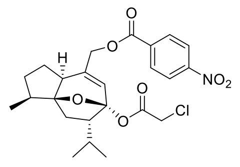	>20	7.18±0.41
1	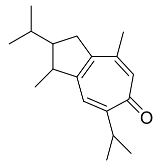	>20	>20
2	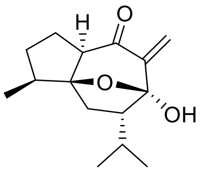	>20	>20
3	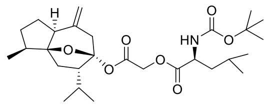	>20	>20
4a	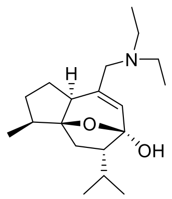	>20	>20
4b	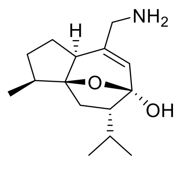	>20	>20

5b	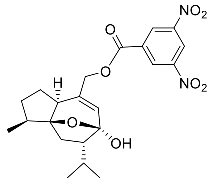	>20	>20
6	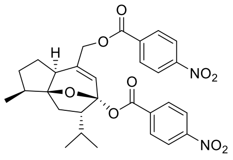	>20	>20

Supplementary Table S2. The sequences of primers used in this study.

Genes	Primer sequence
HMOX1 (HO-1)-Forward	5'-ATGACACCAAGGACCAGAGC-3'
HMOX1 (HO-1)-Reverse	5'-GTGTAAGGACCCATCGGAGA-3'
GAPDH-Forward	5'-GTCTCCTCTGACTTCAACAGCG-3'
GAPDH-Reverse	5'- ACCACCCTGTTGCTGTAGCCAA-3'

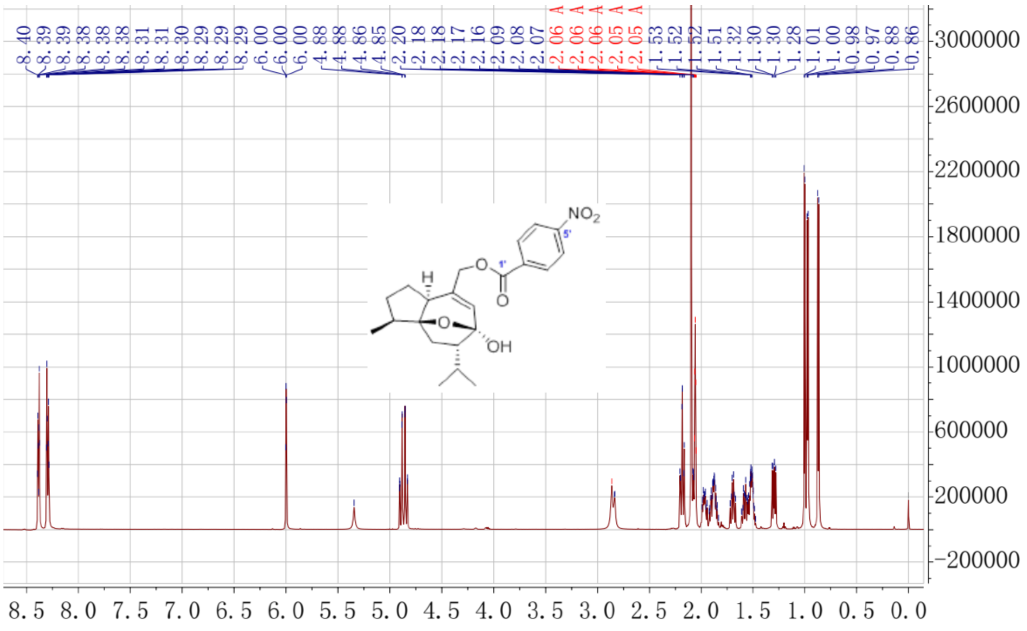

Supplementary Figure S1. 600 MHz 1H NMR Spectrum of HCL-13 in acetone-d6.

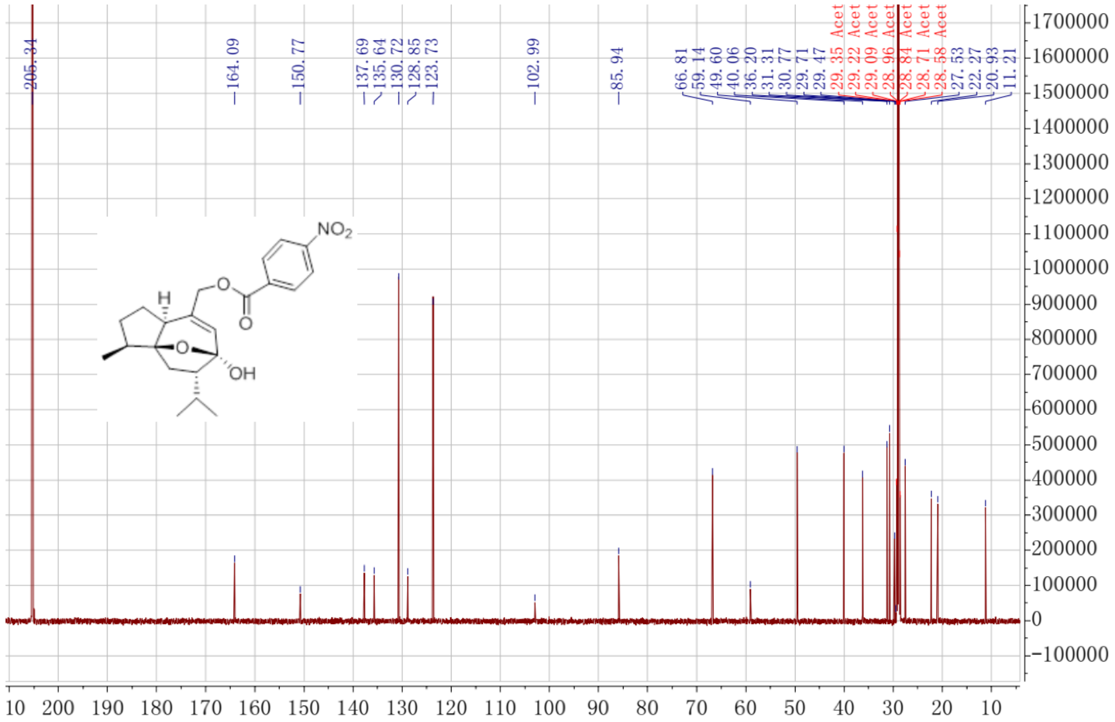

Supplementary Figure S2. 150 MHz 13C NMR Spectrum of HCL-13 in acetone-d6.

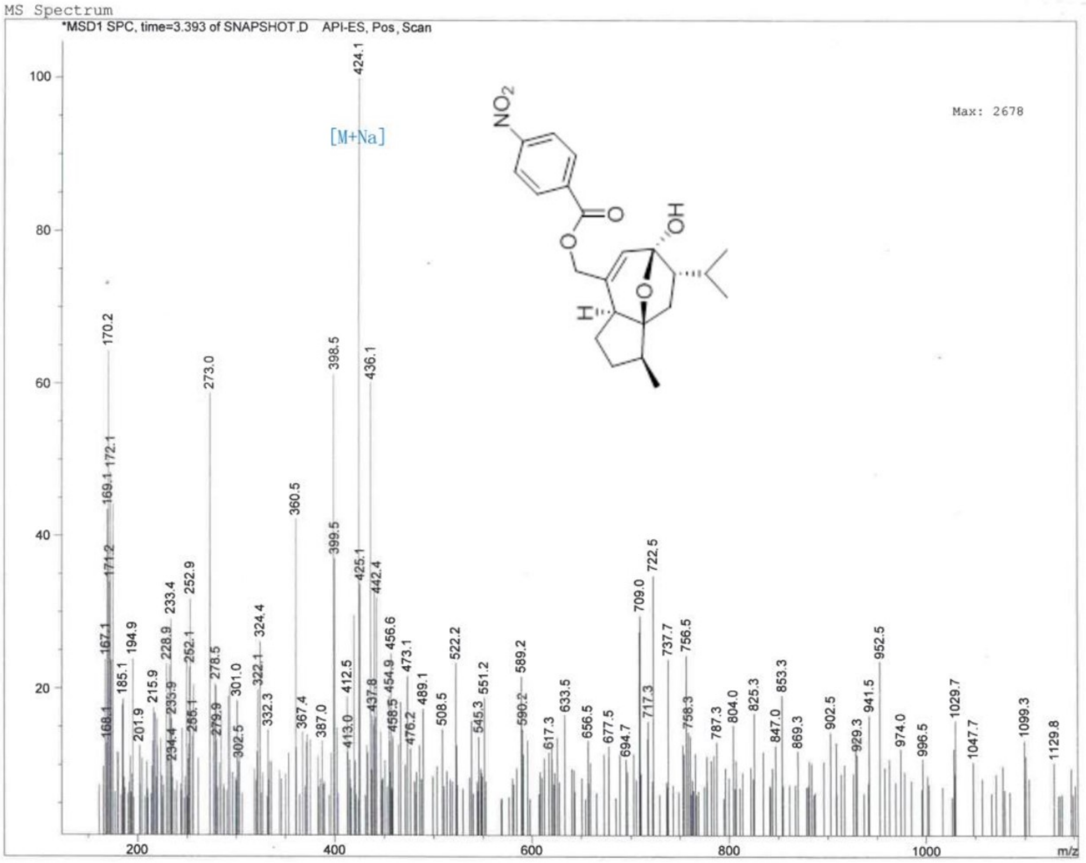

Supplementary Figure S3. ESI-MS of HCL-13.

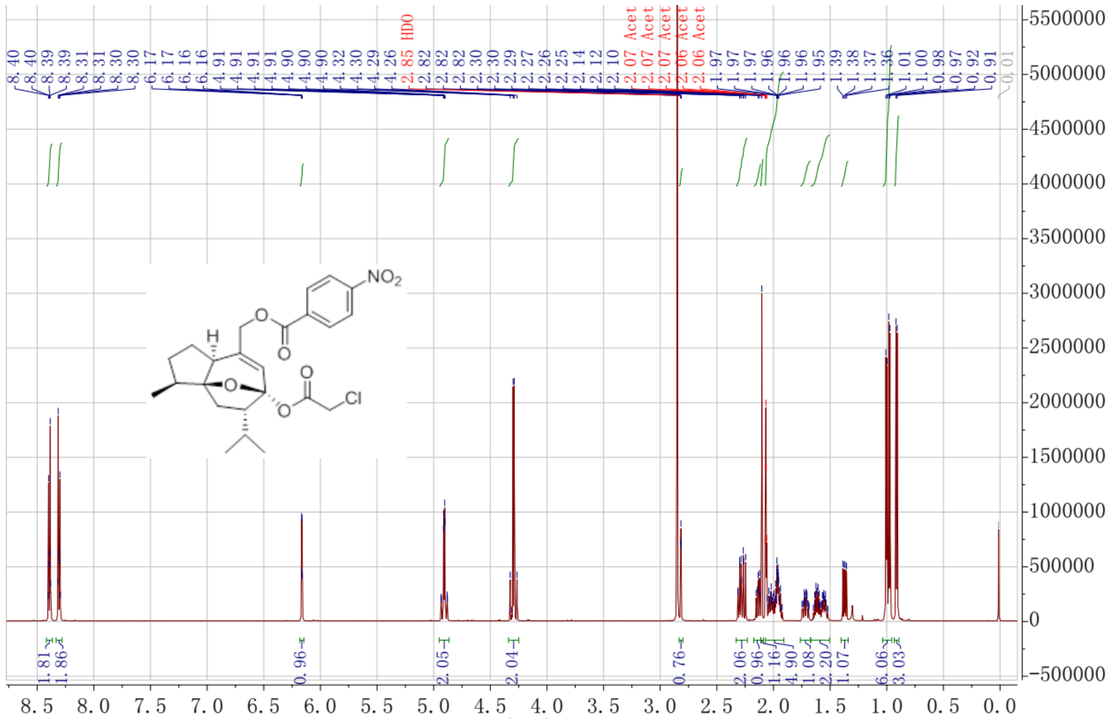

Supplementary Figure S4. 600 MHz ^{1}H NMR Spectrum of HCL-23 in acetone-d_6.

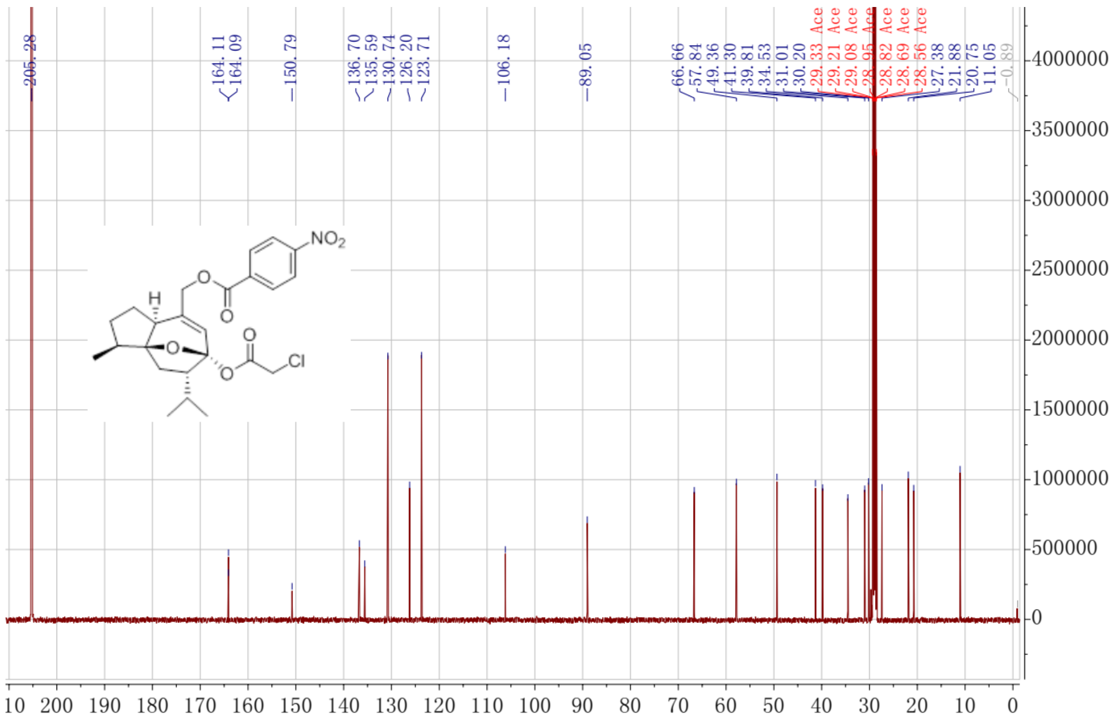

Supplementary Figure S5. 150 MHz ^{13}C NMR Spectrum of HCL-23 in acetone-*d*$_6$.

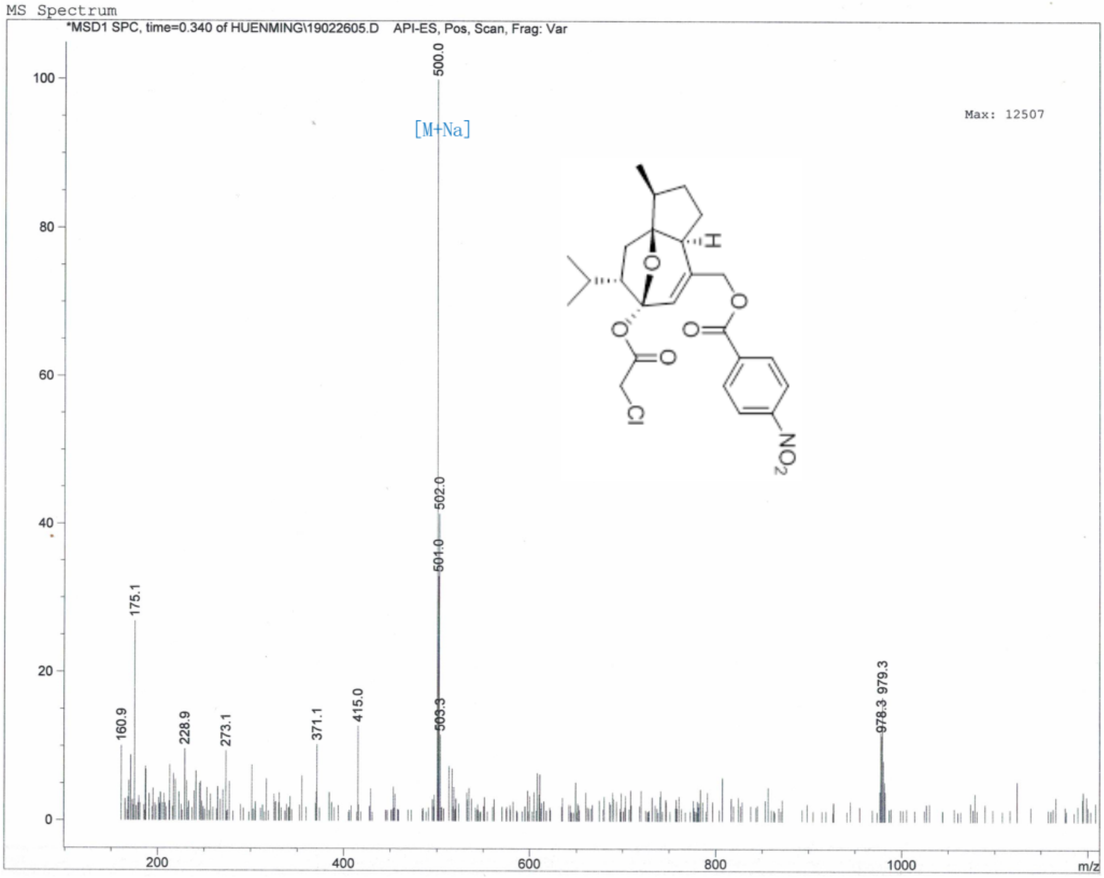

Supplementary Figure S6. ESI-MS of HCL-23.

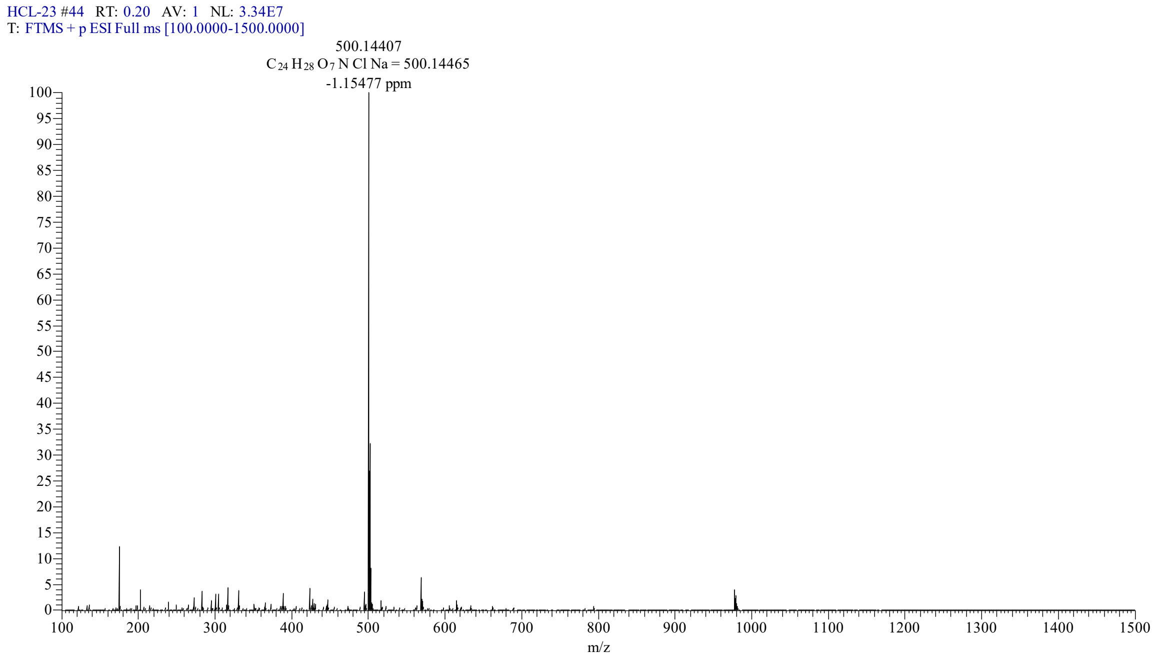

Supplementary Figure S7. HR-ESI-MS of HCL-23.
